# Unlocking Barley’s Phosphorus Efficiency: The Emerging Role of RNA Processing in Low-Phosphorus Adaptation

**DOI:** 10.3390/plants15040547

**Published:** 2026-02-10

**Authors:** Tagarika Munyaradzi Maruza, Muhammad Shahzad, Ameer Khan, Guoping Zhang

**Affiliations:** 1 Department of Agronomy, Key Laboratory of Crop Germplasm Resource of Zhejiang Province, Zhejiang University, Hangzhou 310058, China; tag.maruza@gmail.com (T.M.M.); 12216146@zju.edu.cn (M.S.); ameer_khan11@outlook.com (A.K.); 2 Zhongyuan Institute, Zhejiang University, Zhengzhou 450046, China

**Keywords:** alternative splicing (AS), gene regulatory network (GRN), *Hordeum vulgare*, low-phosphorus adaptation, phosphorus use efficiency (PUE), RNA processing

## Abstract

Improving phosphorus use efficiency (PUE) in crops is critical for sustainable agriculture. Although the transcriptional regulation of phosphate starvation responses, centered on regulators such as the PHR1 and SPX proteins, is well established, the post-transcriptional mechanisms remain incompletely understood. This gap hinders a comprehensive view of how plants adapt to low-P conditions. This review synthesizes current knowledge on the gene regulatory networks involved in low-P adaptation in barley, with a specific focus on the emerging role of RNA processing. It highlights the limited knowledge of how alternative splicing contributes to this response. AS provides a rapid and energy-efficient means of fine-tuning gene expression, expanding proteome diversity and enabling more sophisticated adaptation mechanisms than the relatively binary “on/off” mode of transcriptional control. Several core regulators of AS, including serine–arginine-rich proteins and hnRNPs, have been identified, with the former discussed in detail and illustrated with key examples. Building on the advanced mechanistic insights into AS gained from model crops such as rice, this review proposes a predictive framework to prioritize research targets and experimental strategies. Such an approach can accelerate the discovery of analogous mechanisms in barley, thereby bridging a critical knowledge gap and advancing strategies to improve PUE in this important cereal crop.

## 1. Introduction

The global population is expected to reach 9.7 billion by 2050, driving a 30% to 62% increase in food demand [1,2]. However, scaling up conventional agricultural practices that depend on synthetic fertilizer to meet this demand would intensify environmental degradation. This could exacerbate food insecurity, worsen socio-economic challenges, and heighten vulnerability to climate change impacts [3].

Phosphorus (P), a key component of synthetic fertilizer, is crucial for plants. Playing a vital role in energy transfer, cell membrane structure, and DNA and RNA synthesis in plants, its functions also extend to promoting root development, enhancing flowering and seed production, and improving crop tolerance to abiotic stress. Adequate phosphorus fertilizer can increase yields by 40% to 100% [4,5]. Nevertheless, approximately 70% of arable land in the world suffers from inorganic phosphorus (Pi) deficiency, with this deficiency responsible for up to 40% reduction in productivity globally arable land. This widespread shortage creates acritical reliance on synthetically derived P fertilizers for sustaining crop production [6,7].

P is absorbed by plants as inorganic phosphates (Pi), yet uptake efficiency is remarkably low: only 10–15% of applied P is typically recovered by crops, while the remainder becomes immobilized in the soil or lost through runoff. This unused P contributes to environmental pollution, including heavy metal accumulation and eutrophication, with annual remediation costs estimated at $1 billion and $2.4 billion in Europe and the USA respectively [8,9,10].

More concerning is the finite availability phosphate rock, the primary source of fertilizer P. Current estimates suggest that at present consumption rates, economically viable global reserves may be depleted within approximately 450 years [11]. Studies show that the judicious use of phosphorus fertilizers—tailored to soil Olsen P levels—can help slow the depletion of P reserves while maintaining optimal crop yields [11]. However, achieving sustainable phosphorus management will require a multistakeholder approach that combines such precision application practice with genetic/breeding innovations to enhance P use efficiency [12].

Barley (*Hordeum vulgare* L.) is a versatile and resilient cereal crop of global importance, serving as a key resource for food security, industrial processes, and scientific research. It ranks as the fourth most produced cereal worldwide [13]. While currently used largely as livestock feed, barley holds significant potential for expanded human consumption due to its documented health benefits and resilience under adverse growing conditions, making it a promising crop for addressing future food demands [14,15].

Although modern breeding has successfully enhanced barley’s yield potential, it has also led to a significant narrowing of genetic diversity in commercial cultivars [14,16]. This loss of diversity has consequently reduced their tolerance to abiotic stress [16]. In this context, Tibetan wild barley, the progenitor of cultivated barley, has been identified as a valuable genetic pool harboring novel and useful traits for improving abiotic stress tolerance [17].

While the role of RNA processing during phosphorus (P) deficiency is well-characterized in model crops such as rice and *Arabidopsis*, a comprehensive understanding in barley remains largely elusive. Nevertheless, alternative splicing (AS) has been recognized as an adaptive mechanism in barley under other abiotic stresses, including drought and salinity [18,19]. This strongly suggests that an analogous AS-mediated regulatory layer likely fine-tunes the low-P stress response in barley, especially given studies that have identified AS events occurring under P-deficiency in this crop [20]. The current limited elucidation of AS-mediated low-P adaptation in barley highlights both a critical research gap and a unique research opportunity.

Firstly, this review synthesizes recent advances in the gene regulatory networks (GRNs) governing low-P tolerance in barley, emphasizing the contribution of RNA processing. Second, it leverages this synthesis to propose a focused roadmap for pioneering research on AS-mediated responses to low-P research in barley. The roadmap outlines key steps, including the identification of barley-specific low-P-induced AS events, characterization of resulting gene isoforms and involved AS factors, and ultimately, functional validation aimed at harnessing this regulatoty layer to improve phosphorus use efficiency (PUE).

## 2. Physiological Responses of Barley to Phosphorus Deficiency

Barley’s adaptation to low-P involves a hierarchical resource reallocation strategy that prioritize P acquisition and remobilization at the expense of growth. This strategy results in distinct physiological trade-offs, which are most evident when comparing sensitive and tolerant Tibetan wild genotypes [18]. Key adaptations include a systemic remodeling of root architecture to enhance foraging, a delicate balance of carbon assimilation and energy expenditure, and a sophisticated modulation of hormonal and redox signaling [21,22]. Although transcriptional regulation centered on the PHR1-SPX module is well established, certain physiological changes in plants under low-P stress indicates the presence of rapid molecular changes that extend beyond the transcriptional level [23].

### 2.1. Phosphorus Use Efficiency

Phosphorus use efficiency (PUE), defined as the conversion ratio of total phosphorus input into useful plant biomass, is a key metric for P management in agricultural systems [24]. Enhancing crop PUE represents a viable long-term strategy for promoting environmentally sustainable agriculture [25,26]. As a complex quantitative trait governed by multiple genetic factors, PUE consists of two components: (a) P acquisition efficiency (PAE) and (b) internal P utilization efficiency (PUEi). PAE refers to a plant’s proficiency in foraging and absorbing inorganic phosphate (Pi) from the soil, which is determined by its ability to modify root system architecture (RSA) and to deploy biochemical mechanisms such as exudates and phosphatases [27]. PUEi encompasses the efficiency of internal processes, including the translocation of P from roots to shoots and developing grains, the remobilization of P from senescing tissues, and the metabolic efficiency of P utilization.

### 2.2. Root Remodeling

Plants dynamically remodel their root systems in response to nutrient deficiency [28]. In barley, P deficiency triggers a suite of physiological adaptations, including modification in root morphology, the release of exudates, and the interactions with rhizosphere microbiome [29]. A key morphological adaptation is the inhibition of primary root growth, with simultaneous enhancement of lateral roots and root hairs, collectively serving to expand root surface area and improve soil foraging capabilities [28]. Barley exhibits these typical changes, with studies reporting increased root surface area and the number of root tips in low-P-tolerant genotypes [21,30]. To mobilize organic phosphorus in the soil, barley roots under low-P stress increase the production of exudates (e.g., citrate and malate) and phosphatases [30]. At the molecular level, barley roots under low-P stress undergo a significant reduction in phospholipids, such as lyso- phosphatidylethanolamine (LPE) and lyso- phosphatidylcholine (LPC), with a corresponding increase in Pi-free lipids, a promoter of root hair development [22]. The released Pi from hydrolyzed phosphatidylcholine (PC) and phosphatidylethanolamine (PE) facilitates the production of phosphatidic acid (PA), a promoter of a root hair development. This lipid remodeling alters root cell composition and morphology while helping to maintain P homeostasis [22,31].

### 2.3. Photosynthesis

The availability of phosphorus (P) is fundamental to photosynthetic performance in barley. Under P deficiency, the reduced availability of orthophosphate (Pi) in the chloroplast stroma inhibits the activity of ATP and NADPH synthases. This inhibition leads to proton accumulation and acidification within the thylakoid lumen, which restricts linear electron flow, especially to photosystem I, and ultimately results in severely limited carbon dioxide fixation [32]. Consequently, barley exposed to low-P accumulates 3-phosphoglyceric acid (3PGA), a Calvin cycle intermediate whose further processing requires phosphorylation. The accumulation of 3PGA signifies an impaired capacity to generate the necessary ATP and NADPH under P stress [17]. This reduction in the Calvin cycle’s carbon fixation capacity lowers overall carbon assimilation, leading to decreased plant tillering and biomass [32]. Furthermore, low P levels impede the biosynthesis of chlorophyll and other photosynthetic pigments, reducing light absorption efficiency and exacerbating photoinhibition under high light intensity [33]. P deficiency also disrupts the regeneration of ribulose-1,5-bisphosphate (RuBP), the primary CO_2_ acceptor. As an adaptive strategy, barley upregulates phosphate starvation response genes, such as PHT1 transporters, which redirect energy allocation from photosynthetic processes towards root architectural modification aimed at enhancing P acquisition [17,21,22,30,32].

### 2.4. Energy Redirection and Hormonal Modulation

The reduction in photosynthesis efficiency diminishes the energy available in barley, thereby compromising overall plant growth. This is typically manifested as decreased shoot length, biomass, and tillering under low-P [34]. Although this represents a common response in many crops, certain low-P-tolerant barley genotypes have been reported to maintain or even increase shoot dry weight under low P [35]. While root growth is also inhibited by low-P stress, the extent of retardation is generally less severe than in shoots. This is due to the preferential translocation of carbohydrates to from shoots to roots, resulting in a lower shoot:root ratio and promoting more developed root systems [21,34,35]. Tolerant genotypes, in particular exhibit enhanced root traits such as increased formation of new roots, root hairs, root tips under low-P stress [36]. Under low-P conditions, barley maintains constant P levels in the roots while allowing a reduction in aboveground tissues, thereby conserving this vital element [34].

The response also involves complex phytohormonal modulation. Tolerant barley genotypes exhibit elevated levels of auxins, gibberellic acid and ethylene, hormones associated with root growth, modification and epidermal cell differentiation, respectively [21,30,37]. Moreover, P deficiency promotes the expression of Transport Inhibitor Response 1 (TIR1) in these genotypes, stimulating the degradation of auxin repressor proteins and enabling auxin-response factor 19 (ARF19) to activate genes responsible for lateral root development. Concurrently, cytokinin, a negative regulator to phosphorus starvation response genes, shows decreased levels in the roots of low-P-tolerant barley [21,38,39].

### 2.5. Reactive Oxygen Species

Plants have evolved interconnected regulatory pathways to withstand abiotic stress, though some physiological changes are non-adaptive and merely reflect stress-induced damage [40]. A central aspect of this response is the production of reactive oxygen species (ROS), which function as a double-edged sword: while involved in signaling, their accumulation can be highly detrimental to plant growth. In barley, under different abiotic stresses, including P deficiency, tolerant genotypes mitigate ROS damage upregulating antioxidant enzymes (Table 1) [30,41]. In addition, hormonal crosstalk, particularly between salicylic acid and abscisic acid pathways, has been found to reduce negative stress response in cereals like rice and barley [42].

## 3. Molecular Regulation of Low-P Responses in Barley

**Figure 1:**
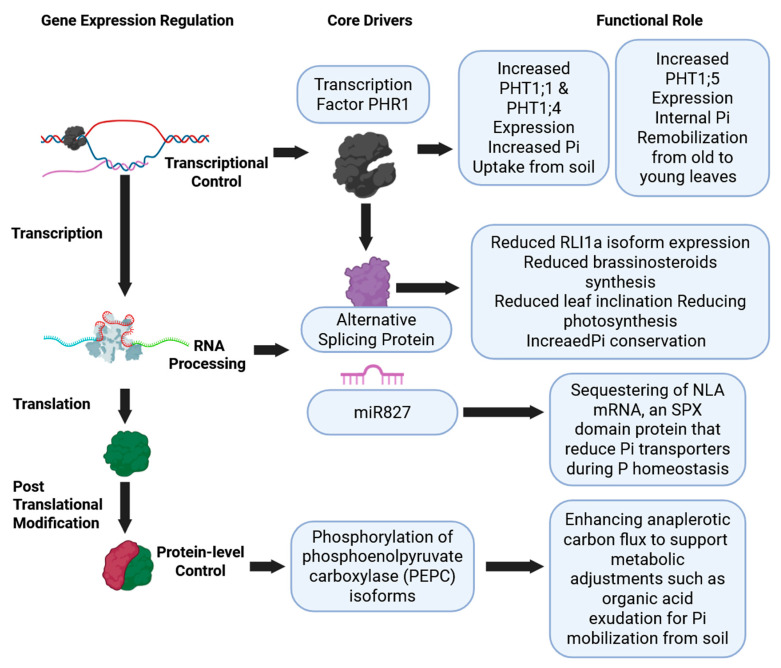
Multilevel regulation of gene expression in response to phosphorus (Pi) availability, spanning transcriptional to post-translational controls. Key drivers, including transcription factors, RNA-binding proteins, microRNAs, and enzymatic modification, mapped to their functional roles in Pi uptake, remobilization, and metabolic adaptation.

Abiotic stresses intensified by climate change, including nutrient deficiency, drought, heat and salinity, significantly constrain plant development and agricultural productivity [40]. To cope, plants rely on multi-layered molecular regulatory systems that orchestrate the above-mentioned physiological responses in barley. Gene regulatory networks (GRNs) form the backbone of the response, operating at multiple levels: first, through transcriptional circuits of TFs and cis-elements; second, via post-transcriptional mechanisms mediated by RNA-binding proteins and non-coding RNAs that control mRNA processing and fate [47,48,49]; and third, through post-translational modifications (PTMs) such as glycosylation, acetylation, phosphorylation, succinylation and ubiquitination, which expand proteomic diversity and refine signaling under [50]. PUE is thus governed by this coordinated GRN, integrating transcriptional, RNA processing and protein-level control (Figure 1).

### 3.1. Transcriptional Control

Transcriptional regulation is the central mechanism of low-P adaptation in plants, primarily orchestrated by transcription factors (TF) that determine the up- or downregulation of target genes. In barley, the MYB transcription factor Phosphate Starvation Response 1 (HvPHR1) acts as a master regulator of P starvation response [51]. Under P- deficiency, HvPHR1 is released from inhibition by SPX proteins, allowing it to bind to the P1BS cis-element (GNATATNC) in the promoters of P starvation-induced (PSI) genes (Table 2) [52,53].

These PSI targets include phosphate transporter (PHTs), miRNAs and other factors such as Phosphate Transporter Traffic Facilitator 1 (PHF1) and Induced by Phosphate Starvation 1 (IPS1). PHR1 possesses several homologs, including PHL1, PHL2 and PHL3 that exhibit functional redundancy as well as the formation of homo- and heterodimers with PHR1, which enables their regulatory functions [54,55]. Under low Pi, PHR1 is released from SPX-mediated inhibition and activates PSI genes, thus orchestrating root remodeling, P uptake, and internal P remobilization. While this PHR1-centered module is evolutionarily conserved, barley-specific targets or splice variants may fine-tune its activity [52].

**Table 2:** PSI genes regulated by PHR1 in cereal crops.

PSI Gene	Regulatory Role/Function	Source
*HvSPDT/OsSPDT/AtSPDT*	Encodes a Pi/H+ plasma membrane co-transporter responsible for phosphate uptake from the soil.	[56]
*PHO1;1*	Reloads Pi to the phloem of diffuse vascular bundles in nodes for root to shoot translocation	[35,56]
*PHO1;2*	Unloads Pi from the xylem of enlarged vascular bundles in node facilitating the distribution of developing grains	[35,52,56]
*PHO1;3*	Mediates remobilization of phosphate under very low-P regimes	[35,52,56]
*PHO1;7*	Expresses in phloem of vascular bundles; proposed role in P redistribution and anther development	[35,52,56]
*RNS1*	Encodes a ribonucleae that degrades extracellular RNA, which is a potential source of Pi. This represents a form of Pi scavenging	[57]
*IPS1*	Contains a sequence with a mismatched non-coding RNA motif that is complementary to miR399, and sequesters miR399 finetuning the suppression of PHO2	[52,54]
*PAP12, PAP26*	Encodes purple acid phosphatase (APase) isoenzymes secreted into soil to mobilize organic phosphorus	[22]
*EXPA8, RSL4*	Promotes root structure architecture (RSA) remodeling via hair elongation/growth,	[58,59]
*SPX-MFS1/3*	Functions as vacuolar Pi efflux transporters, crucial for the remobilization of stored P	[56]
*BFN1*	A bifunctional nuclease that degrades nucleic acid during senescence, facilitating internal phosphorus remobilization	[60]
*SQD2*	Replaces membrane phospholipids with sulfolipids under low-P stress, conserving phosphorus for essential metabolic processes	[22]
*DREB1C*	A transcriptional integrator that promotes the expression of phosphate starvation-induced (PSI) genes	[61]

Beyond transcriptional control, reversible epigenetic modifications, such as DNA methylation, regulate gene accessibility during abiotic stress [62]. Under low-phosphorus stress increased methylation in transposable elements (TEs) regions correlates with a decrease in small interfering RNAs (siRNAs), with tolerant genotypes exhibiting fewer differentially methylated regions (DMRs) [63]. For instance, in soybean, methylation of the gene body of *GmSCL9*, a negative regulator of gibberellic acid signaling that suppresses root growth [63], suggest how DNA methylation can serve as an adaptive mechanism to modulate transcription during Pi deficiency.

### 3.2. RNA Processing

Under Pi deficiency, plants dynamically regulate RNA processing to fine-tune gene expression and optimize Pi acquisition and utilization [4]. A key mechanism involves miRNA-mediated post-transcriptional regulation. Specifically, miR399 and miR827 mediated RNA interference of HvPHO2 (PHOSPHATE 2) and NLA (NITROGEN LIMITATION ADAPTATION) transcripts, respectively. These genes encode E3 ubiquitin ligases that, under phosphate-replete conditions, tag HvPHT1 phosphate for degradation, thereby limiting Pi uptake. Their downregulation under low-Pi stress stabilizes HvPHT1 proteins, enhancing barley’s phosphate acquisition [51,64].

In addition to regulating uptake, RNA processing also facilitates internal phosphorus remobilization. *RNS1* (Ribonuclease 1), a phosphorus starvation-induced gene (PSI), degrades RNA to liberate Pi for reallocation to essential processes [57]. Manipulating these RNA-level mechanism, such as expression miR399, miR827, or *RNS1* offers a promising strategy for engineering crops with improved PUE, which could reduce agricultural dependence on phosphate fertilizers.

Alternative splicing (AS) is an important RNA processing mechanism that enables rapid adaptation to plant stress within hours. Approximately 80% of multiexon genes in plants produce multiple isoforms [65]. Serine/arginine-rich (SR) and heterogeneous ribonucleoprotein (hnRNP) are key protein families that regulate AS, providing a fast and energy-efficient means of adjusting gene expression under stress conditions [66]. For instance, Studies [67] have identified the SR protein OsSCL26 in rice, which regulates P-response related genes. Knockout mutants of *OsSCL26* showed altered transcript ratios of *OsSPX-MFS2*, a dual-domain Pi transporter/sensor and OsNLA, a ubiquitin-linked modulator of Pi/N homeostasis, revealing the role of *OsSCL26* in splicing-mediated P adaptation.

### 3.3. Protein-Level Control

This regulatory process involves post-translational modifications (PTM) that rapidly modulate protein activity and stability to either optimize or disrupt barley’s P acquisition and utilization. HvSPX proteins (named after SYG1, Pho81 and XPR1) function by binding phosphorylated inositol to sense cellular Pi status [22]. Under high Pi conditions, SPX proteins, such as SPX1 and SPX4 interact with PHR1 transcription factors, sequestering them in the cytoplasm and preventing their binding to P1BS motifs in the promoters of PHT1and other Pi-starvation-induced (PSI) genes, thereby repressing transcription [22]. Further fine-tuning of P uptake is achieved through PHO2/NLA-mediated ubiquitination and subsequent proteasomal degradation of HvPHT1 transporters. Additionally, phosphorylation by MAPK3/6 and CDPKs regulates PHT1 activity: MAPK3/6 promotes endocytosis under high-Pi conditions, while CDPKs modulate transporter function during stress. Under Pi deficiency, SPX proteins also inhibit TOR kinase, reallocating resources from growth to phosphorus recycling through processes such as RNS1-mediated RNA decay [68]. Collectively, these PTM-driven mechanisms allow plants to dynamically adjust P acquisition and remobilization in response to changing environmental P availability.

## 4. The Emerging Role of RNA Processing in Plant Stress Adaptation

Transcriptional control has long been recognized as a primary mechanism in plant stress responses; however, it provides an incomplete picture of the sophisticated regulatory landscape required for adaptation. A more dynamic and energy-efficient layer of regulation occurs at the post-transcriptional level through RNA processing. By acting on pre-existing mRNAs, this process enables plants to rapidly modulate protein output, increase proteome diversity, and conserve energy under fluctuating environmental stresses, such as low-P stress. Growing evidence indicates transcriptional control and RNA processing functioning as complementary and hierarchical systems. Deeper understanding of this post-transcriptional dimension of gene regulatory networks (GRN) is essential for a holistic view of barley adaptation to low-P stress.

### 4.1. The Roles of RNA Processing in Fine-Tuning Stress Adaptation in Plants

While extensive attention has been devoted to studying transcriptional control, a primary and well-established mechanism in plant stress adaptation, RNA processing remains comparatively understudied. However, stress adaptation is a multilayered phenomenon, and transcriptional control alone provides an incomplete picture. RNA processing reveals additional regulatory layers that are essential for a comprehensive understanding of the molecular mechanisms underlying adaptation of plants to abiotic stresses [69,70,71,72].

Transcriptional control involves chromatin remodeling, recruitment of transcription machinery, and transcription factor binding, processes that are relatively slow compared to RNA processing, which acts on pre-exiting mRNA by activating, silencing or altering it in response to stress [73]. RNA processing operates closer to the functional gene product, enhancing the speed, precision, and spatial specificity of adaptation [71,74]. Moreover, while transcriptional regulation often functions in a binary on/off manner, RNA processing enables nuanced, reversible fine-tuning. This is exemplified by the generation of upstream open reading frames (uORFs) by through transposon-derived sequences, which precisely modulates protein output and influences mRNA function, localization, stability or interaction partners [70].

RNA processing also offers energy-efficient regulation. Under phosphorus deficiency, global transcriptional reprogramming is energetically costly, whereas RNA processing allows recycling of existing transcripts, conserving ATP [75,76]. Furthermore, RNA processing interfaces with epigenetic markers to contribute to stress memory, priming plants for recurrent stress through heritable RNA changes [77].

Another distinctive feature of RNA processing is its mobility: RNA processing components can move cell-to-cell via plasmodesmata (cell to cell) and systemically through the phloem, enabling cross-tissue regulation, though larger complexes such as spliceosomes and polyadenylation machinery are generally immobile [78,79]. Recent studies showed that transcriptional control and RNA processing function as a complementary and hierarchical regulatory system [69]. While transcriptional studies remain essential for understanding gene activation, research into RNA processing deepens our insight into how plants achieve complex, precise and reversible adaptation under economically significant abiotic stresses such as phosphorus deficiency [71,72].

Alternative splicing (AS) is a key post-transcriptional mechanism that enables plants to rapidly and efficiently adapt to environmental changes by generating multiple protein isoforms from a single gene [67]. Under abiotic stress, AS significantly contributes to plant survival by expanding proteome diversity and enabling precise physiological adjustment [67,80]. AS modulates a broad of stress-responsive pathways, including ABA signaling [80], the expression of stress-related transcription factors [81], the biosynthesis of antioxidant and secondary metabolites [82], and the regulation of ion transport [67].

**Figure 2:**
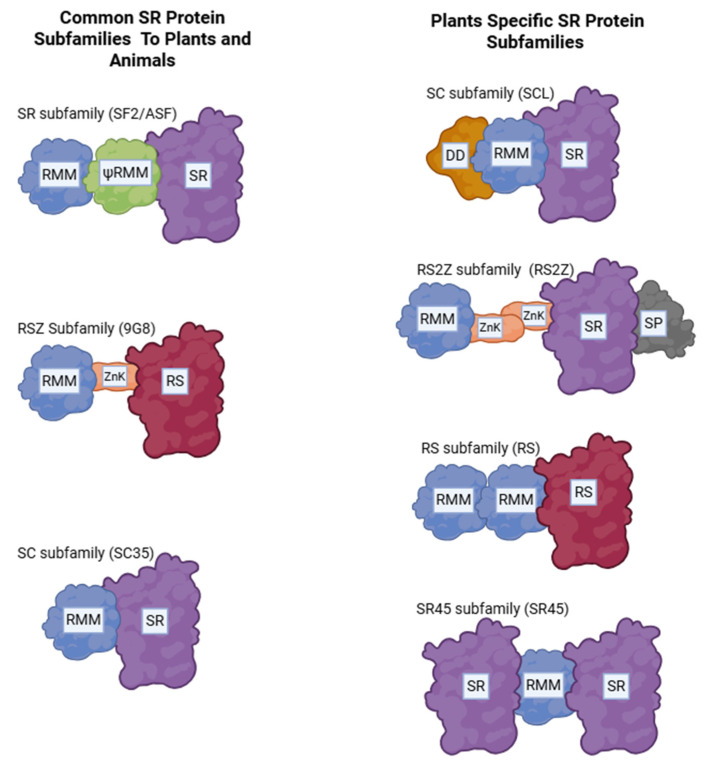
Schematic of SR proteins found in plants and animals. RRM and ψRRM are the RNA recognition motifs with ψRRM containing a SWQDLKD motif: This domain (usually 1 or 2 copies at the N terminus) recognizes and binds specific sequences in pre-mRNA that is to undergo splicing. SR represents the serine–arginine dipeptide-containing domain: it serves the role of protein–protein bridging in the spliceosome, splice site activation and regulation via phosphorylation. RS represents domains rich in arginine and serine: this domain mediates protein–protein interactions like other SE proteins or spliceosomal component like U1-70K or U2AF as well as protein–RNA interactions. A heavily phosphorylated domain which regulates SR protein localization to the nuclear speckle. ZnK denotes CCHC-type zinc knuckles: This domain enhances RNA binding in combination with the RRMs, contributing to the specificity of certain RNA motifs. SP denotes a domain rich in serine and proline: This often-auxiliary domain likely contributes to the structural flexibility as well as protein–protein interactions. DD denotes rare domains: These domains include glycine/arginine-rich prio-like domains, N-terminal extensions or acid extensions, which play various roles in RNA binding, phase separation and protein–protein interactions.

The regulation of AS is carried out by splicing factors, such as RNA-binding proteins or spliceosome components, that recognize specific cis-regulatory sequences in pre-mRNA, direct spliceosome assembly, and determine splice site selection [81]. These factors are categorized according to their functional roles in splicing and stress responses, with major classes including serine/arginine-rich (SR) proteins, heterogeneous nuclear ribonucleo-proteins (hnRNPs), and other RNA-binding proteins [80]. Among these, SR proteins are the most extensively studied AS regulators. They participate in both constitutive and alternative splicing, and are characterized by the presence of at least one RNA-recognition motif (RRM) at their N-terminus and arginine and serine dipeptide-rich (RS) C-terminal domain [59,83]. SR proteins are further subdivided into distinct subfamilies based on shared structural features, as summarized in Figure 2.

### 4.2. Alternative Splicing in Barley

Like many other crops, barley employs alternative splicing (AS) to rapidly adapt to abiotic stress. A seminal genome-wide analysis revealed that AS is a pervasive feature of the barley transcriptome, with 51% of genes undergoing splicing events, predominantely through intron retention, highlighting a substantial intrinsic capacity for transcriptome plasticity [84]. This study further revealed that under salt stress barley generates AS isoforms of SQUAMOSA Promoter Binding Protein-like (SPL) transcription factors (*SPL2*, *SPL10* and *SPL11*), which are targets of miR156 and miR157. This finding highlights a key node of crosstalk within a complex regulatory network. The interplay between AS and miRNA pathways allows for sophisticated, multilayer control of gene expression, which are essential for stress adaptation. Typically, SPLs promote the transitions from juvenile to the adult phase; under stress, however, miRNAs inhibit their activities to facilitate adaptive responses such as increased tillering, altered root structure and metabolic adjustments [85]. Additionally, network analysis revealed an interaction hub centered on core splicing components, such as U1-70K and various serine/arginine-rich proteins, with key miRNA biogenesis genes such as *DCL1* and *HEN1* also undergoing AS. This indicates a functional feedback loop between the splicing machinery and miRNA regulatory pathways.

This was experimentally validated using the elongation factor family protein gene (*MLOC_3412*), demonstrating that AS in barley is dynamic and regulated process, with specific isoforms being favored under adverse conditions. Under salinity stress, a tolerant barley accession displayed alternative splicing, with genotype-like XZ26 exhibiting 40 and 38 salt-responsive differentially expressed genes that also underwent alternative splicing (AS-DEGs) in the roots and shoots, respectively [18]. These AS-DEGs were enriched for functions in ion transporters and transcription factors, a regulatory layer proposed to enhance and amplify K^+^/Na^+^ homeostasis.

Drought stress has also been found to trigger AS-mediated adaptation in barley through the production of a splice variant of the light-harvesting complex gene *HvLHCA4.2*, designated *LHCA4.2b*, This variant is exclusively expressed in drought-tolerant barely genotypes during the recovery period, suggesting a two-phase adaptation strategy: an immediate drought stress response to drough stress followed by active recovery [19]. The study demonstrated that *LHCA4.2b* plays a dual role in enhancing drought-tolerance. First, it stabilizes Photosystem I/Light-Harvesting Complex I (PSI-LHCI) super complex through strengthening interaction interfaces with LHCA1, thereby improving energy transfer efficiency and directly reducing ROS generation at the source. Second, it fine-tunes ABA signaling by upregulating transcription of the positive regulators *ABF1/ABF3*, while suppressing repressors *ABI3/ABI4/ABI5*, thereby promoting stomatal closure.

The mechanistic principles of AS-mediated adaptation to salt and drought stress strongly support its relevance in low-P adaptation, as ion homeostasis, ROS management and energy-efficiency resource reallocation, processes finely tuned by AS, alre also central components of the plant response to phosphorus deficiency [20].

### 4.3. Alternative Splicing in LP Adaptation

Limited research has been conducted on the role of AS in barley under low-P stress. The available literature, based on comprehensive full-length transcriptome analysis, has revealed that barley possesses two or more isoforms for 25,810 genes, suggesting that AS is a fundamental mechanism for generating proteomic complexity [20]. However, this study did not perform an integrated analysis to directly identify which specific differentially expressed genes (DEGs), particularly those with greater expression diversity in tolerant genotypes across treatments, are involved in critical processes such as phospholipid degradation, phosphate transport and phosphorylation/dephosphorylation pathways. This foundational work establishes AS as a key component of the molecular strategy for P efficiency. The scarcity of functional data in barley necessitates a comparative approach with model crops in which AS-mediated low-P-adaptive mechanisms have been identified. These systems are not used as direct templates, but serve as valuable sources of mechanistic principles and candidate regulatory genes.

Alternative splicing has been identified as a mechanism contributing to low-P stress adaptation in rice, although this field remains relatively underexplored [67,83]. A key example is the REGULATOR OF LEAF INCLINATION 1 (RLI1) gene in *Oryza sativa*, which exhibits a dual functional role depending on its splicing pattern under low P conditions [83]. Under low-P stress, overall *RLI1* transcription decreases, yet expression of RLI1b’s isoform, containing both a MYB domain and a coiled-coil (CC) domain) is favored over the RLI1a isoform, which contains a MYB DNA-binding domain. Functionally, RLI1b acts as a phosphate response regulator (PHR2), modulating Pi starvation signaling and Pi homeostasis, whereas RLI1a activates genes involved in brassinolide (BL) biosynthesis and signaling, thereby coordinating both plant growth and Pi starvation signaling [83,86]. The CC domain facilitates the formation of RLI1 homo- or heterodimers, enhancing their binding affinity to P1BS motifs in the promoters of PSI genes. This shift in the RLI1b-to-RLI1a ratio is not merely a corrective response, but a mechanism of significant physiological importance, illustrating how AS can rapidly redirect the proteome from growth-oriented processes to stress adaptation. Notably, the Pi starvation-induced erect leaf phenotype in rice, which reduces light interception and photosynthetic activity to conserve Pi, is associated with the reduced expression of the RLI1b isoform [83].

In *Arabidopsis thaliana*, genome-wide transcriptional analysis identified a splice variant of PHO2 under low-P conditions. This isoform lacks the miR399-binding site, introducing a key regulatory layer that fine-tunes ion that P starvation response and prevents over-accumulation of phosphate transporters and other PSI proteins [87]. Highly tissue-specific AS patterns have also been observed in *Solanum lycopersicum* (tomato) under low-P conditions. Gene ontology analysis revealed that the differentially spliced genes were primarily involved in mRNA processing, U2-type pre-spliceosome assembly, and nucleotide binding in both root and shoot tissues. Among these, splicing regulators such as SR protein genes, particularly Solyc089069120 showed differential intron retention across Pi treatments. The finding suggests that Pi availability influences the splicing patterns of splicing regulators, which in turn alter the transcript sequence of their target PSR genes, ultimately shaping the crop’s adaptation to P deficiency [88].

Furthermore, the SR protein OsSCL26, located on the third chromosome of *Oryza sativa*, modulates the expression of multiple PSI genes, including *OsPHT1;1*, *OsPHT1;8*, *OsPHT2;1*, *OsPHT4;3*, *OsSPX-MFS1*, *OsSPX-MFS2*, *OsNLA2* and *OsSPX2*. In CRISPR/Cas-9-mediated *OsSCL26* knockout mutants, the abundance of *OsSPX-MFS2.1* decreased, while abundance of OsSPX-MFS2.2, OsNLA2.1 and OsNLA2.2 increased compared to the wild type, indicating that OsSCL26 regulates P homeostasis at the post-transcriptional level by modulating splicing within the miR827-NLA regulatory network [67]. Beyond Pi deficiency, AS also contributes broadly to abiotic stress adaptation, as summarized in Table 3.

In barley, direct functional studies linking specific AS events to low-P response remain scarce; however, the evidence from genome-wide full-length transcriptome profiling reveals extensive and dynamic AS events during both the P-deficiency and P-resupply phases [20]. These AS events are widespread across chromosomes 2, 3, 5 and 7, strongly suggesting their impact on genes involved in crucial processes such as phosphate metabolism, root system remodeling and stress signaling [20,89,90].

This post-transcriptional regulation likely supports key physiological adaptations in barley. For instance, low-P conditions trigger root remodeling and enhance the expression of P transporters (PHT1 family) and PSI genes to improve P acquisition efficiency (Section 2.2). AS may fine-tune isoforms of transporter, regulatory factors such as homologs of OsSCL26, an SR protein that modulates splicing of SPX-MFS and NLA genes involved in Pi uptake and remobilization, or lipid metabolism enzymes, thereby enabling faster responses than transcriptional changes alone. Similarly, impaired photosynthesis and energy redirection under low P (Section 2.3 and Section 2.4), including reduced Calvin cycle efficiency, preferential carbohydrate allocation and hormonal modulation, may be modulated by AS-generated isoforms of photosynthetic regulators, hormone signaling components or energy metabolism genes [91].

In tolerant barley genotypes, AS likely helps maintain ROS homeostasis (Section 2.5) by producing stress-specific isoforms of antioxidant enzymes (e.g., those listed in Table 1). This complements transcriptional mechanisms centered on PHR1/SPX (such as miRNA regulation, RNA degradation) as described in Section 3.2, while enabling nuanced, reversible finetuning of the proteome to integrate root foraging, internal P remobilization and stress mitigation. Beyond Pi deficiency, AS also contributes broadly to abiotic stress adaptation in barley and related cereals, as summarized in Table 3.

**Table 3:** Splicing factors involved in abiotic stress adaptation.

Alternative Splicing Factor	Species	Role/Function	Source
OsSCR106	*Oryza sativa*	Regulates pre-mRNA splicing, particularly alternative 3′-splice site (A3SS) selection, for a large set of genes under stress and normal conditions.	[66]
SR45	*Arabidopsis thaliana*	Different isoforms have specific roles: SR45.1 in flower development and SR45.2 in root growth. Also influence pre-mRNA.	[66,92,93]
RS33	*Oryza sativa*	regulates pre-mRNA splicing in response to abiotic stresses.	[59,66]
RS40, RS41, SCL30a	*Arabidopsis thaliana*	Loss of function mutants display hypersensitivity to salt and ABA stress.	[66]
SR34b	*Oryza sativa*	Loss of function mutant displayed a shorter root phenotype and higher cadmium accumulation in response to cadmium treatment.	[66]
AtSF1	*Arabidopsis thaliana*	Alternative splicing of FLOWERING LOCUS M (FLM), this links it to temperature response regulation.	[59]
OsSCL26	*Oryza sativa*	Regulates phosphorus homeostasis by influencing absorption and distribution through dual role of transcription and splicing of P transport genes.	[67]
OsSCL25	*Oryza sativa*	Regulates P uptake and accumulation in shoots; conserved function in regulating genes involved in P distribution in shoots.	[67]

RNA processing provides multiple advantages for plants responding to abiotic stress, yet alternative splicing stands out due to several distinctive features. Unlike mRNA decay and nonsense-mediated decay (NMD), which primarily regulate mRNA abundance and turnover, AS enables the fine-tuning of pre-existing pre-mRNA, not only removing certain transcripts but also enhancing proteome diversity, thereby supporting more complex adaptative responses [80].

Compared to other RNA processing mechanisms such as siRNA-guided DNA methylation and silencing, AS operates on a much faster timescale. As a co-transcriptional process, AS can generate adaptative protein variants within seconds or minutes. In contrast, siRNA-mediated silencing involves multiple sequential steps, including siRNA biogenesis, RISC loading, target recognition, DNA methylation, and eventual silencing, a process that typically requires hours or days to complete [94]. Functionally, siRNA-guided silencing serves as a long-term strategy for maintaining genomic integrity by permanently silencing transposable elements, whereas AS acts a rapid tactical response to dynamic environmental conditions [95]. Moreover, several AS factors play dual roles, functioning not only in splicing but also in transcriptional regulation. For example, the splicing factor OsSCL26 regulates the alternative splicing of genes such as *OsGI*, *OsFTL4*, and *OsBEIa*, and has been validated to mediate splicing of phosphorus-responsive genes like *OsSPX-MFS2* and *OsNLA2* under low-P conditions. In addition to splicing, OsSCL26 also upregulates the expression of multiple P-related genes, including *OsPHT1;1*, *OsPHT1;8*, *OsSPX-MFS1*, *OsSPX-MFS2*, *OsNLA2* [67]. Moreover, under sufficient P supply, *OsSCL26* knockout mutants exhibit reduced shoot height compared to the wild-type plants, suggesting an additional role for this gene in normal growth and development [67].

A similar dual functionality has been observed with the AS factor OsSCR106, which is essential for abiotic stress adaptation in rice. Transcriptomic analysis revealed that OsSCR106 regulates distinct sets of genes at the transcriptional and splicing levels, functioning both as a splicing factor and a transcription factor [66]. Likewise, the SR protein SR45, an ortholog of human RNPS1, plays a dual role as a splicing factor and a participant in transcriptional regulation through chromatin remodeling [92].

This multifunctional capacity makes AS factors attractive candidates for identifying master regulatory hubs and associated quantitative trait loci (QTLs) involved in phosphorus stress response [66,67,92].

### 4.4. Divergent Adaptation Strategies: Imperatives for Dryland Cereals in Low-Phosphorus Research

The distinct physiological and molecular strategies adopted by semi-aquatic and dryland cereals under low-P stress highlight the need to investigate these complex, polygenic traits [96]. Semi-aquatic crops like rice, which evolved in flooded soils where P is largely fixed in insoluble forms, prioritize biochemical mechanisms for P solubilization, including the exudation of organic acids and phosphatases [22,56]. In contrast, dryland cereals like barley, adapted to well-aerated soils where P availability is limited mainly by diffusion, have evolved more pronounced morphological adaptations, such as extensive remodeling of root system architecture and enhanced root hair proliferation, to improve soil foraging capacity [21,97].

These divergent physiological strategies are governed by distinct molecular cue. Although conserved P starvation response (PSR) regulators such as PHR1 operate across species, their downstream targets and regulatory networks are fine-tuned by ecological niche [98]. The emerging role of RNA processing, particularly alternative splicing (AS), introduces another layer of divergence. For example, AS factors, such as SR proteins, include family members that have evolved specialized roles in particular lineages [66]. Relying solely on models from semi-aquatic crop or Arabidopsis risks overlooking these critical, ecologically specific adaptations. Hence, an appropriate dryland cereal crop model is required to account for the evolutionary divergence in low-P adaptation between dryland and semi-aquatic cereals.

Barley, with its well-characterized diploid genome, offers a practical advantage over wheat, which has a large and complex hexaploid genome. This diploid structure simplifies gene function and inheritance studies by reducing complication from multiple similar gene copies [99].

## 5. Conclusions and Future Perspectives

Phosphorus use efficiency (PUE) is a critical determinant of agricultural productivity and environmental sustainability. As research has shown, plant adaptation to low-P stress is governed by a multitiered gene regulatory network (GRN). Considerable progress has been made in elucidating transcriptional roles of central regulators such as PHR1 and SPX proteins in economically important crops like rice, maize, wheat and barley [22,100].

Translation of emerging insights into RNA processing and AS-mediated low-P adaptation into practical crop improvement requires leveraging natural genetic variation, particularly from wild resources or landraces such as Tibetan wild barley. These germplasms harbor superior low-P tolerance traits, including enhanced root plasticity, efficient Pi remobilization, and the maintenance of P homeostasis under P deficiency [22,35].

To effectively apply knowledge of AS-mediated low-P stress response from well-studied crops like rice to barley improvement, molecular biologists can adopt comparative and functional genomics pipelines [101]. Such studies should prioritize ortholog identification, transcriptome mining, and targeted validation. Key insights from rice and Arabidopsis, such as the splicing factors summarized in Table 3, provide strong templates, as demonstrated in prior studies [66].

The first step involves identifying barley orthologs of well-characterized AS regulators and low-P responsive genes using sequence similarity tools (against barley reference genome Morex V3) and phylogenic analysis. This should be followed by full-length long-read RNA sequencing on platforms such as PacBio Iso-Seq across contrasting genotypes [20,71] to detect dynamic AS events in these orthologous genes under low-P conditions. Focus can then be directed to isoforms that correlate with tolerant traits, such as enhanced root remodeling, elevated Pi transporter activity or improved ROS homeostasis. Candidate AS events and factors can be validated through isoform-specific qPR-PCR and/or CRISPR-based editing. Finally, integrating validated marker or edited alleles into breeding program via marker-assisted selection (MAS) using Tibetan wild barley or landraces donors or through direct introgression, will accelerate the development of high-PUE cultivars with improved performance under low-P conditions. This ortholog-driven approach bridges mechanistic knowledge from model species with barley’s genetic resources, offering a cost-effective route to trait enhancement while reducing reliance on trial and error functional studies.

The identification of AS factors in barley offers a strategic opportunity to improve this fourth most important cereal crop, while also advancing the broader understanding of AS-mediated low-P tolerance. Further research into the rapid and energy efficient mechanism of AS is expected to reveal novel targets for breeding and bioengineering. Uncovering these sophisticated post-transcriptional regulatory layers will enable PUE optimization through faster and metabolically efficient strategies [102].

As a dryland crop, barley employs AS factors and regulatory mechanisms that may be more directly applicable to other dryland cereals than those identified in semi-aquatic species such as rice. This is especially relevant for traits such as root system architecture [97]. While AS under low-P stress has been investigated in rice, studies in dryland cereals remain scarce. Therefore, the exploration and characterization of AS in cultivated and wild barley represent a strategic pathway to improving PUE across vital dryland cereal crops.

## Figures and Tables

**Table 1:** Antioxidant enzymes in barley with upregulation under abiotic stress.

Enzyme	Function of Enzyme	Source
Glutathione-S-Transferase (GST)	Scavenges ROS protecting plant from damage ROS inflict on key cellular structure and molecules.	[30]
Isoflavone Reductase (IFR)	Acts as an antioxidant in response to reactive oxygen species (ROS) during root development.	[35]
Glutamine Synthetase	Catalyzes component required for respiration process and tricarboxylic acid (TCA) cycle, involved in stress defense.	[30]
Methionine Sulfoxide Reductase	Reduces methionine sulfoxide back to methionine, protecting proteins from oxidative damage.	[30]
Superoxide Dismutase (SOD)	Acts as the first line of defense against ROS by catalyzing the dismutation of the superoxide radical (O_2_•^−^) into oxygen (O_2_) and hydrogen peroxide (H_2_O_2_).	[41]
Catalase (CAT)	A crucial enzyme that detoxifies hydrogen peroxide (H_2_O_2_) by breaking it down into water (H_2_O) and oxygen (O_2_), preventing oxidative damage.	[41]
Aldehyde Oxidase (AO)	A molybdenum-containing enzyme involved in the biosynthesis of abscisic acid (ABA) and the production of reactive oxygen species (ROS) like H_2_O_2_ and O_2_•^−^, which can act as signaling molecules to trigger defense responses.	[41,43,44]
Ascorbate Peroxidase (APX)	A key component of the ascorbate–glutathione cycle that uses ascorbate to reduce H_2_O_2_ to water, playing a vital role in managing ROS levels within chloroplasts and other cellular compartments.	[45]
Aldose reductase	Converts toxic aldehydes (generated by ROS-induced lipid peroxidation) to less reactive alcohols.	[46]
Aldo-keto reductase	Detoxifies reactive carbonyl species produced by oxidative stress.	[46]
Dihydroflavonol 4-reductase	Key enzyme in the flavonoid/anthocyanin biosynthesis pathway. Flavonoids are potent antioxidants.	[46]

## Data Availability

No new data were created or analyzed in this study.
